# Integration of psychiatry into palliative care units: Retrospective analysis of psychiatric consultation reasons and diagnoses by age and gender, with a treatment overview

**DOI:** 10.1097/MD.0000000000044754

**Published:** 2025-09-19

**Authors:** Emre Vuraloglu, Mehmet Atilgan

**Affiliations:** a Department of Family Medicine, Kirşehir Training and Research Hospital, Kirşehir, Turkey; b Department of Psychiatry, Kirşehir Training and Research Hospital, Kirşehir, Turkey.

**Keywords:** palliative care, referral and consultation, tertiary care centers

## Abstract

The objective of this study was to investigate psychiatry consultations for patients in a palliative care unit, comparing consultation reasons and diagnoses after psychiatrists’ consultations according to age and gender, and to describe the treatments provided by psychiatrists. This study, designed as a retrospective, descriptive, and cross-sectional, involved 97 patients who were hospitalized in the palliative care unit of Turkey Kirsehir Training and Research Hospital between September 2023 and September 2024. Data were obtained from hospital medical records. Statistical analyses were performed to compare the clinical characteristics of the patients, consultation reasons, and diagnoses after psychiatrists’ consultations according to age and gender. Statistical analyses were performed using IBM SPSS Statistics version 20.0, and a *P*-value <.05 was considered statistically significant. The most common reason for palliative care unit consultation to psychiatry was agitation, and the most common diagnosis made by psychiatrists was sleep disorders. The most frequently used treatment for sleep disorder is quetiapine (83.3%). A statistically significant difference was identified in anxiety diagnoses between female and male patients (*P* = .004), with higher rates observed in females. Delirium diagnosis was found to be significantly more prevalent in patients aged ≥65 years compared to those aged 18 to 64 (*P* = .025). Also, non-psychiatric organic causes (pain, dyspnea, fatigue, loss of appetite etc) were significantly more common in males than in females (*P* = .027) and in patients aged 18 to 64 than in patients aged ≥ 65 (*P* = .019). The main conclusion of this study can be summarized as including psychiatrists in palliative care unit team or communicating with them to ensure effective treatment for diagnoses such as sleep disorders, delirium, anxiety disorders, and depression, which are commonly encountered in palliative care units and can be interpreted as early signs of worsening patient prognosis.

## 1. Introduction

Palliative care is a comprehensive, multidisciplinary approach.^[1]^ The World Health Organization defines palliative care as follows: Actions and strategies to improve the quality of life and of death for older people with a serious illness or who are reaching the end of their lives by preventing or relieving physical, psychological, social, and spiritual suffering for themselves and their families, including regular assessment and management.^[2]^

Patients facing serious, life-limiting illnesses frequently experience a high burden of psychiatric comorbidities, which can profoundly affect a range of clinical and psychosocial outcomes. These conditions, particularly depressive disorders and anxiety are known to negatively impact not only the patient’s quality of life but also their prognosis, symptom burden, and overall healthcare utilization. Despite their significant consequences, research indicates that psychiatric comorbidities are often underrecognized and inadequately treated in palliative care populations.^[3–5]^

The collaboration between psychiatrists and palliative care unit (PCU) physicians is crucial for the early identification and management of depression, anxiety, hopelessness, grief, disorientation, and for enhancing patients’ quality of life.^[6]^ Currently, psychiatrists are becoming more involved in palliative care. The national cross-sectional study carried out in the United Kingdom in 2005 revealed that access to a psychiatrist in hospices was 30%; however, a subsequent survey conducted 15 years later indicated an increase to 80%.^[7]^ Building on this context, the objective of the present study was to conduct a detailed investigation of psychiatry consultations for patients in a PCU. Specifically, the study aimed to compare consultation reasons and the diagnoses made after psychiatrists’ consultations according to age and gender, and to provide a descriptive analysis of the treatments administered by psychiatrists, to highlight the importance of psychiatric involvement in palliative care settings.

## 2. Methods and materials

### 2.1. Study design and hospital setting

This is retrospective, descriptive, and cross-sectional study. This study was conducted in Kirsehir Training and Research Hospital. Kirsehir Training and Research Hospital is a tertiary-level healthcare facility situated in Kirsehir, Turkey, which includes a 20-bed in the PCU. The PCU team comprises a family medicine specialist, a general practitioner, a gerontologist, a physiotherapist, 11 nurses, 5 healthcare officers, 5 clinical support personnel, and 4 cleaning staff members. In Turkey, palliative care is not recognized as a distinct medical specialty.

### 2.2. Participants and sample

The study population consisted of patients who were treated at the PCU of Kirsehir Training and Research Hospital between 15 September 2023 and 15 September 2024. No randomization was used in the selection of patients. All patients who met the exclusion and inclusion criteria of the study participated in the study and formed the sample of the study.

The criteria for inclusion were as follows: The patient’s age should be 18 years and older. Patient must have been treated in the PCU of Kirsehir Training and Research Hospital between 15 September 2023 and 15 September 2024. The patient must have received at least one psychiatric consultation after hospitalization in the PCU.

Exclusion criteria were as follows: The patient has a psychiatric illness at the time of hospitalization in the PCU and the patient is under 18 years old.

A total of 97 patients were evaluated in this study by rigorously applying the predefined inclusion and exclusion criteria within the specified time frame.

### 2.3. Data collection

The demographic and clinical characteristics of the patients were analyzed, including age, gender, duration of hospitalization in the PCU, feeding style, tracheostomy status, presence of pressure ulcers, and reasons for hospitalization in the PCU. These were assessed at the time of the first psychiatric consultation, except for the duration of hospitalization, which was obtained from patient files closed due to death or discharge.

In this study, the reasons for the first psychiatric consultation for each palliative care patient and the psychiatrists’ responses to this consultation were investigated. In cases where a patient had more than one psychiatric consultation, only the first consultation was included in the evaluations.

The electronic medical information system of Kirsehir Training and Research Hospital was utilized as the study’s data source. The researchers did not interfere with the file information during the data collection and patient evaluation process. Patient information was kept private. The researchers only accessed the specified variables.

### 2.4. Statistical analysis

The statistical measures presented include means, standard deviations, medians, minimums, and maximums for continuous variables, while frequencies and percentages are reported for discrete variables. To determine whether continuous variables followed a normal distribution, the Kolmogorov–Smirnov test was applied. Comparisons between groups were conducted using non-parametric Mann–Whitney *U* tests for continuous variables, comparing mean age between males and females and mean duration of hospitalization between patients aged 18 to 64 years and those aged ≥65 years, and either the χ^2^ or Fisher exact test for categorical data (psychiatric consultation reasons and diagnoses after psychiatrists’ consultations according to gender and age group). A power analysis using G*Power was performed to determine the sample size required for statistical significance in assessing differences in consultation reasons and post-consultation psychiatric diagnoses according to age and gender. Power analysis confirmed that the sample size was adequate to achieve statistical significance. Statistical procedures were carried out using IBM SPSS Statistics software version 20 (IBM Corp., released 2011). A *P*-value of <.05 was considered statistically significant, with a 95% confidence interval.

### 2.5. Ethical consideration

Ethical approval for the study was obtained by the Ethics Committee of Kirsehir Ahi Evran University Faculty of Medicine Health Sciences. (Decision No: 2024-18/159, Date: 05/11/2024).

## 3. Results

A total of 97 consultations with palliative care patients were recorded during the study period. Demographic data, clinical features, and reasons for hospitalization in the PCU are shown in Table 1. The mean age of the patients was 72.82 ± 15.71 years. The mean age of women was 75.85 ± 12.12 years and that of men was 69.98 ± 18.13 years (*P* = .210) (Mann–Whitney *U* test). Most patients were aged 65 and over. The mean period of hospitalization in the palliative care unit was 11.35 ± 10.28 days. The mean period of hospitalization for patients aged 18 to 64 years was 9.19 ± 9.35 days, while the mean period of hospitalization for patients aged ≥65 years was 11.95 ± 10.50 days (*P* = .060) (Mann–Whitney *U* test). The feeding style of 85.6% of the patients was enteral. 6.2% of the patients had tracheostomy. In 42.3% of patients, pressure ulcers were present. Musculoskeletal disorders, neoplasms, and heart disease were the most common reasons for PCU admissions.

**Table 1:** Demographic characteristics, clinical features, and reasons for hospitalization of the patients.

n = 97	Mean ± SD Median (Min–Max)	
Age (yr)	72.82 ± 15.71 76 (24–97)	
Duration of hospitalization in palliative service (d)	11.35 ± 10.28 7 (1–60)	
	n	%
Age groups		
18–64 yr	21	21.6
≥65 yr	76	78.4
Gender		
Female	47	48.5
Male	50	51.5
Feeding style		
Oral	54	55.7
Nasogastric	17	17.5
Percutaneous endoscopic gastrostomy	12	12.4
Parenteral	14	14.4
Tracheostomy		
No	91	93.8
Yes	6	6.2
Pressure ulcer		
No	56	57.7
Yes	41	42.3
Reasons for hospitalization in palliative service		
Lung disease		
No	84	86.6
Yes	13	13.4
Ischaemic & non-iscaemic heart disease		
No	75	77.3
Yes	22	22.7
Cerebrovascular diseases		
No	81	83.5
Yes	16	16.5
Dementia		
No	84	86.6
Yes	13	13.4
Renal failure		
No	80	82.5
Yes	17	17.5
Congenital malformations		
No	88	90.7
Yes	9	9.3
Musculosketal disorders		
No	68	70.1
Yes	29	29.9
Protein energy malnutrition		
No	80	82.5
Yes	17	17.5
Malignant neoplasms		
No	73	75.3
Yes	24	24.7
Other		
Yes	3	3.1

**Figure 1.**
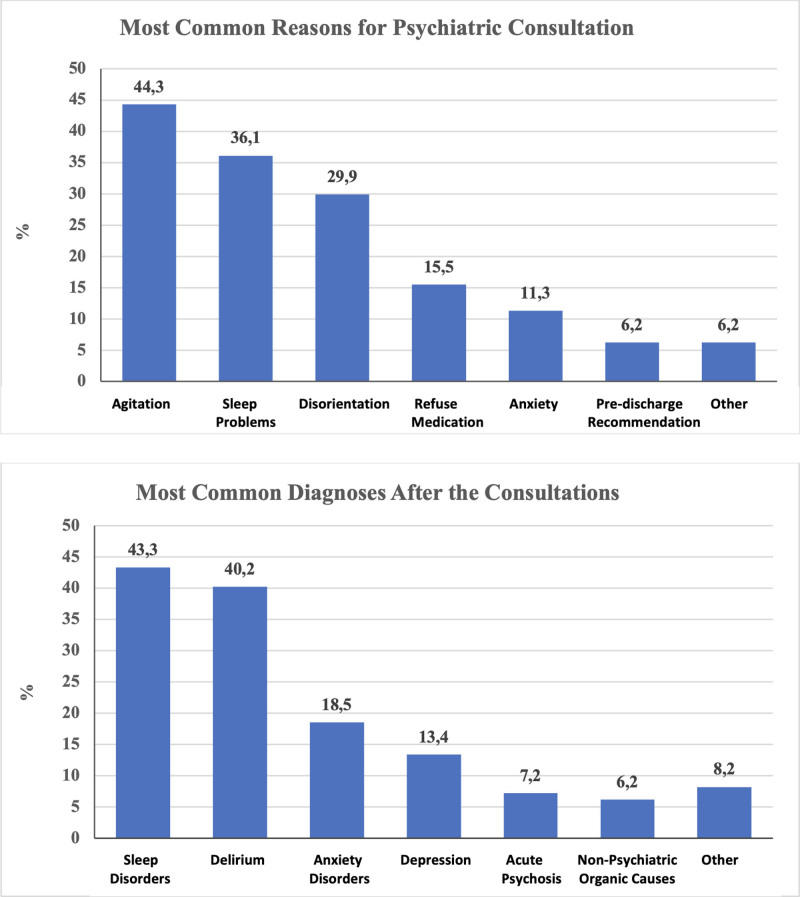
The most common reasons for psychiatric consultations among palliative care patients and the most common diagnoses made by psychiatrists following these consultations.

Figure 1 shows the most common reasons for psychiatry consultations in palliative care patients and the most common diagnoses made by psychiatrists after consultations. The most common reason for consultation was agitation. The most common diagnosis made by psychiatrists was sleep disorders.

In Table 2, the relationship between the reasons for consultation of palliative care patients and the demographic data of the patients is examined. There was no significant difference (*P* > .05) in the reasons for consultation between female and male patients, nor in the reasons for consultation between patients aged 18 to 64 years and those aged ≥65 years (*P* > .05). (χ^2^ test/Fisher exact test).

**Table 2:** The relationship between the most common reasons for psychiatric consultation and demographic data.

Most common reasons for psychiatric consultation	Gender			Age group		
	Female	Male	*P*	18–64 yr	≥65 yr	*P*
	**n (%**)	**n (%**)		**n (%**)	**n (%**)	
Disorientation						
No	31 (66.0)	37 (74.0)	.387	17 (81.0)	51 (67.1)	.220
Yes	16 (34.0)	13 (26.0)		4 (19.0)	25 (32.9)	
Agitation						
No	26 (55.3)	28 (56.0)	.946	11 (52.4)	43 (56.6)	.732
Yes	21 (44.7)	22 (44.0)		10 (47.6)	33 (43.4)	
Sleep problems						
No	32 (68.1)	30 (60.0)	.407	14 (66.7)	48 (63.2)	.767
Yes	15 (31.9)	20 (40.0)		7 (33.3)	28 (36.8)	
Refusal of medication						
No	41 (87.2)	41 (82.0)	.476	20 (95.2)	62 (81.6)	.179
Yes	6 (12.8)	9 (18.0)		1 (4.8)	14 (18.4)	
Anxiety						
No	41 (87.2)	45 (90.0)	.668	17 (81.0)	69 (90.8)	.246
Yes	6 (12.8)	5 (10.0)		4 (19.0)	7 (9.2)	
Pre-discharge consult.						
No	44 (93.6)	47 (94.0)	1.000	19 (90.5)	72 (94.7)	.607
Yes	3 (6.4)	3 (6.0)		2 (9.5)	4 (5.3)	
Other						
No	44 (93.6)	47 (94.0)	1.000	19 (90.5)	72 (94.7)	.607
Yes	3 (6.4)	3 (6.0)		2 (9.5)	4 (5.3)	

* *P* < .05 was accepted as the limit of statistical significance at a 95% confidence interval (χ^2^ Test/Fisher Exact test).

Table 3 analyzes the relationship between the diagnoses made by psychiatrists after consulting with palliative care patients and their demographic characteristics. A statistically significant discrepancy was identified between the anxiety diagnoses of female and male patients (*P* = .004). Specifically, anxiety diagnosis rates were found to be higher among female patients compared to male patients. A statistically significant discrepancy was observed in the rates of delirium diagnosis among patients aged 18 to 64 years and those aged ≥65 years (*P* = .025). The rate of delirium diagnosis was found to be higher in patients aged 65 years and over. Moreover, our study data showed that non-psychiatric causes were significantly more common in males than in females (*P* = .027) and in patients younger than 65 years of age than in patients aged 65 and over. (*P* = .019) (χ^2^ test/Fisher exact test).

**Table 3:** **The relationship between the most common diagnoses after psychiatrists’ consultations and demographic data**.

Diagnoses after the consultations of psychiatric specialist	Gender			Age group		
	Female	Male	*P*	18–64 yr	≥65 yr	*P*
	**n (%**)	**n (%**)		**n (%**)	**n (%**)	
Depression						
No	39 (83.0)	45 (90.0)	.310	19 (90.5)	65 (85.5)	.728
Yes	8 (17.0)	5 (10.0)		2 (9.5)	11 (14.5)	
Anxiety disorders						
No	34 (72.3)	47 (94.0)	.004	17 (81.0%)	64 (84.2%)	.744
Yes	13 (27.7)	3 (6.0)		4 (19.0%)	12 (15.8%)	
Sleep disorders						
No	26 (55.3)	29 (58.0)	.790	15 (71.4)	40 (52.6)	.124
Yes	21 (44.7)	21 (42.0)		6 (28.6)	36 (47.4)	
Delirium						
No	26 (55.3)	32 (64.0)	.384	17 (81.0)	41 (53.9)	.025
Yes	21 (44.7)	18 (36.0)		4 (19.0)	35 (46.1)	
Acute psychosis						
No	44 (93.6)	46 (92.0)	1.000	19 (90.5)	71 (93.4)	.643
Yes	3 (6.4)	4 (8.0)		2 (9.5)	5 (6.6)	
Non-psychiatric organic causes						
No	47 (100)	44 (88.0)	.027	17 (81.0)	74 (97.4)	.019
Yes	-	6 (12.0)		4 (19.0)	2 (2.6)	
Other						
No	45 (95.7)	44 (88.0)	.270	17 (81.0)	72 (94.7)	.064
Yes	2 (4.3)	6 (12.0)		4 (19.0)	4 (5.3)	

* *P* < .05 was accepted as the limit of statistical significance at a 95% confidence interval (χ^2^ test/Fisher Exact test).

Table 4 shows the diagnoses and treatments given to palliative care patients by psychiatrists after consultation. Since psychiatrists can make more than one diagnosis and prescribe more than one treatment for a patient, the data are presented as numbers and percentages rather than statistical relationships between diagnosis and treatment. The most frequently used drugs in psychiatric diagnoses were as follows: Quetiapine for sleep disorders (83.3%), haloperidol for delirium (92.3%), haloperidol for anxiety disorders (55.5%), selective serotonin reuptake inhibitors for depression (84.6%), and risperidone for acute psychosis (57.1%).

**Table 4:** The most common medical regimens according to diagnosis after psychiatric consultation.

	Depression (n = 13)	Anxiety disorders (n = 18)	Sleep disorders (n = 42)	Delirium (n = 35)	Acute psychosis (n = 7)	Non-psychiatric organic causes (n = 6)
**Treatment**	**n (%**)	**n (%**)	**n (%**)	**n (%**)	**n (%**)	**n (%**)
SSRI	11 (84.6)	5 (27.7)	9 (21.4)	3 (7.7)	1 (14.3)	–
SNRI	1 (7.7)	–	–	–	–	–
Mirtazapine	2 (15.4)	4 (22.2)	5 (11.9)	1 (2.6)	–	–
Quetiapine	5 (38.5)	7 (38.8)	35 (83.3)	16 (41.0)	2 (28.6)	–
Haloperidol	2 (15.4)	10 (55.5)	17 (40.5)	36 (92.3)	1 (14.3)	1 (16.7)
Olanzapine	1 (7.7)	4 (22.2)	4 (9.5)	–	3 (42.9)	–
Risperidone	–	1 (5.5)	–	1 (2.6)	4 (57.1)	–
Drug-free follow up	–	–	1 (2.4)	–	–	5 (83.3)

SNRI = Serotonin–norepinephrine reuptake inhibitor, SSRI = Selective serotonin reuptake inhibitor.

## 4. Discussion

This study retrospectively examined the reasons for psychiatric consultations in the PCU, the distribution of diagnoses, and their relationship with demographic characteristics. Our findings revealed that the most common reason for consultation was agitation and the most common diagnosis made by psychiatrists was sleep disorders. When evaluated according to gender, it was found that anxiety was diagnosed at a significantly higher rate in female patients compared to male patients. In the comparison between age groups, delirium was significantly more common in the ≥ 65 age group than in the 18 to 64 age group. Moreover, referrals due to non-psychiatric indications were found to be significantly more common among male patients compared to females, and among individuals younger than 65 years compared to those aged 65 and above.

In our study, the most common reason for consultation was agitation. Similar to our research, in a study conducted by Hendriks et al with 372 nursing home patients in The Netherlands, the most common symptom was agitation, found in more than half of the patients.^[8]^ Delirium is the predominant cause of agitation in hospitalized patients, followed by dementia and psychosis in diminishing order of prevalence. Numerous factors, including pain, anxiety disorders, epilepsy, and personality problems, contribute to the genesis of agitation via frontal lobe dysfunction.^[9]^ In our study, the frequency of delirium and additional diseases in the etiology of agitation may be compatible with this finding. Agitation may be detected and treated more frequently because it is easily noticed by the patient’s caregivers/the treatment team and negatively affects the patient’s compliance with treatment.

According to this study, the second most common reason for consultation by PCU doctors was sleep problems; the most common diagnosis by psychiatrists was sleep disorders. Sleep disorders, including insomnia, have been observed in a range of 60 to 90% of PCU patients and negatively correlated to age and positively correlated with complaints of depression and anxiety.^[10,11]^ To sum up, sleep disorders are the most prevalent diagnosis, despite variations in frequency, according to the parallel findings of the aforementioned studies and our own. Although sleep disorders were the most common diagnosis in our study, the use of different scales or diagnosis by clinical interview, evaluation of sleep complaints as symptoms or disorders, and disease distributions in palliative units may explain the higher frequencies in the mentioned studies.

Although there are physiological problems in sleep with age, the majority of studies show that there is no significant correlation.^[12–15]^ In our study, sleep disorders were observed more frequently in patients aged 65 years and older compared to those under 65; however, this difference did not achieve statistical significance. This finding may be influenced by the small sample size, variability in underlying medical conditions, and the limitations associated with a cross-sectional study design.

Delirium was the second most common diagnosis made by psychiatrists in our research. Similar rates were reported in the following studies. A systematic review by Watt et al indicated that delirium is diagnosed at a rate of 42% during patient admission in PCUs and is observed in almost all patients before death.^[16]^ Moreover, a study including 350 PCU patients revealed a delirium rate of 44%.^[17]^ Our analysis revealed a statistically significant difference in the frequency of delirium diagnoses between patients aged 18 to 64 and those aged 65 and above, with a higher prevalence observed in the ≥65 age group. In a systematic review conducted in 2021, which included 9 studies investigating delirium risk factors in palliative care patients, a notable relationship was found between risk factors such as being male, older age and cachexia.^[17]^ Additionally, the increase in delirium with advancing age, as frequently reported in the literature was found to be consistent with and statistically significant in our study.^[17,18]^ The increase in the frequency of comorbidity diagnoses with advancing age, the use of drugs that can cause delirium, and malnutrition due to malignancies may play a major role in the elevation of delirium prevalence in older patients. Early recognition of delirium and training of the treatment team/caregivers against delirium symptoms may reduce patient mortality and treatment costs.

A statistically significant discrepancy was identified between the anxiety diagnoses of female and male patients in our study. Specifically, anxiety diagnosis rates were found to be higher among female patients compared to male patients. A consistent finding emerged in the research conducted by Donnelly et al, Kirkova et al, and Zimmerman et al, indicating a consensus that anxiety is more common among terminally ill women.^[19–21]^ However, contrary to this, Carey et al discovered that anxiety levels were lower in women than in men in their study, which included 172 cancer patients.^[22]^ Furthermore, a meta-analysis encompassing 4007 palliative care patients across 7 countries reported an overall anxiety disorder prevalence of 9.8%, with no statistically significant variations according to age or gender.^[23]^ Anxiety, which is detected in a wide range in palliative care samples and worsened by factors such as pain, delirium, sepsis, dyspnea, etc, increases the risk of functional deterioration.^[24]^ The reasons for this wide spectrum in anxiety frequency may include applying strict criteria for anxiety disorder or using anxiety symptoms as criteria, the distribution of psychotic/consciousness-affecting illnesses in the sample, and the expression of anxiety through somatic complaints in societies with low cultural emotional expression.

Although depression is generally more prevalent among women in the overall population, studies have reported comparable rates between men and women in terminally ill patients, and in some cases, a higher incidence has been observed in men.^[25–27]^ A major reason for this is that being dependent on others for basic needs such as eating, dressing, and using the toilet is a risk factor for depression in men.^[26]^ In large-scale studies conducted on PCU and terminal stage patients, the prevalence of depression was found to be in a wide range.^[28,29]^ The 14.3% prevalence of depression identified in this study appears lower than that reported in other studies; however, this may be attributed to the subtler clinical presentation of depressive symptoms compared to conditions like delirium and agitation, the reduced likelihood of consultation for such symptoms, and their frequent misattribution to physical complaints such as fatigue. Depression has been associated with deterioration in functionality, heightened pain, and poor prognosis.^[29]^ Physicians must be careful to distinguish neurovegetative symptoms of depression from symptoms related to the patient’s medical condition, such as fatigue, weight and appetite loss, diminished energy, impaired attention, and sleep problems.^[30]^

The diagnosis of non-psychiatric organic causes in our study includes complaints such as pain, dyspnea, fatigue, sleep disorders and loss of appetite, which are thought to have organic etiology rather than psychogenic, and since they are few, they were collected under this heading. The findings of our study indicated that non-psychiatric organic etiologies were significantly more prevalent in male patients compared to females, and in individuals under the age of 65 compared to those aged 65 and above. Since psychiatric conditions such as depression, anxiety disorder, physical illnesses or drug treatments may have varying degrees of share in these complaints, care should be taken in differential diagnosis. While studies showing that fatigue is more common in females, there are also studies that say the opposite.^[22,31]^ In addition, there are studies showing that dyspnea and cancer pain are more common in males than females.^[32,33]^ On the contrary, in a study of 31,771 Danish palliative care cancer patients, pain was found to be more common in women.^[34]^ All things considered, in terms of non-psychiatric organic causes, there is uncertainty about gender differences when looking at symptom clusters rather than specific symptoms in the literature.

In terms of gender difference, in a study examining 710 adult inpatients, it was found that there was inequality in care between men and women; moreover, women had to complain of symptoms more often to be considered.^[35]^ In our study, the significant increase in non-psychiatric organic causes in men can be explained by the difficulty and rarity of reporting in women, the differences in the distribution of diagnoses between men and women, and the evaluation of symptoms as a group. Although there is more data showing that non-psychiatric organic causes decrease with age, there are also studies showing the opposite. In a study conducted with 1000 patients, Walsh et al found that many complaints, such as pain, nausea, etc, were more common in young patients than in the elderly.^[36]^ Moreover, in a study including 1032 terminal PCU patients, a decrease in pain, dyspnea, fatigue, and sleep problems was found with advancing age.^[37]^ On the contrary, Hansen et al showed that loss of appetite and fatigue increase with age.^[34]^ One possible explanation is that older individuals may exhibit greater life satisfaction, demonstrate more acceptance of their symptoms, and be less inclined to report them.^[38]^ It has also been suggested that patients under the age of 65 tend to express their symptoms more frequently, potentially because older adults often attribute issues such as pain or fatigue to normal aging processes.^[35]^ Similarly, Gott et al showed that patients were consulted less with increasing age.^[39]^ The other reasons why symptoms are reported more frequently in younger patients include the deterioration of family and economic conditions due to the risk of death, difficulty in coping with and accepting terminal illnesses because of the destruction of future dreams, and the psychological and physical burden brought by all these processes.^[40]^ In conclusion, the reasons explaining our data, which are parallel to the dominant literature data, are that cognitive impairment with advancing age reduces symptom reporting and, in addition, the older patient group attributes their symptoms to the natural aging process compared to younger patients.^[41,42]^

In the treatment section of our study, the most commonly used drugs in psychiatric diagnoses were as follows: Quetiapine for sleep disorders, haloperidol for delirium, haloperidol for anxiety disorders, selective serotonin reuptake inhibitors for depression, and risperidone for acute psychosis. Studies have indicated that benzodiazepines play a significant role in the management of short-term insomnia in terminally ill patients. Trazodone, mirtazapine, and quetiapine are utilized in the management of sleep disorders, with careful consideration of their adverse effects.^[43]^ Except for the use of benzodiazepines, the treatments in our study were consistent with the literature. Benzodiazepine use is limited due to the increased risk of delirium, the risk of respiratory depression, the availability of ampoule forms only in our hospital, and the difficulties experienced in prescribing. Lorazepam, which we used frequently in outpatient and inpatient psychiatry practice in Turkey until 2 years ago, has been discontinued in our country.

A review by Grassi et al in 2015 demonstrated that first-generation antipsychotics and second-generation antipsychotics exhibited equivalent efficacy in treating delirium.^[44]^ In a study conducted with 240 inpatient hospice patients, haloperidol was identified as the most frequently administered medication for managing delirium.^[45]^ Our findings align with these previously reported results. Additionally, quetiapine was the second most common use in delirium, which was not surprising considering the agitation and sleep problems common in delirium. In addition to psychotropic treatment, solutions to problems that cause or worsen delirium, such as pain, dehydration, malnutrition, constipation, sensory problems, immobility, sleep problems, and lack or excess of stimuli, should be considered in delirium management.^[46,47]^

Selective serotonin reuptake inhibitors were found to be the most common drug class in the treatment for depression in our study, as mentioned in the following studies, in accordance with the literature. A PCU guideline and a review have recommended the use of selective serotonin reuptake inhibitors, serotonin and norepinephrine reuptake inhibitors and mirtazapine in the treatment of depression.^[29,48]^ Since the effects of these drugs appear within 2 to 4 weeks, there are also studies recommending the use of methylphenidate and ketamine in cases where rapid effects are desired. Among these agents, methylphenidate may be effective in alleviating fatigue; however, it should be administered with caution in individuals with cardiac conditions, reduced appetite, or insomnia. Ketamine also has anti-anxiety and anti-suicidal effects but can cause dissociative complaints.^[29,49]^ Since methylphenidate is a red prescription (controlled) drug in our country and only approved for the attention deficit hyperactivity disorder indication, it cannot be used in the treatment of depression. Ketamine has been approved as a depression drug in Turkey but is not covered by reimbursement. Due to these problems, these drugs are not used in the treatment of depression in our country. If appropriate indications and reimbursement are provided, it will increase the comfort of life of patients with the rapid antidepressant effect required, especially in short-term PCU patients.

In the treatment of anxiety, it was determined that quetiapine, haloperidol, and selective serotonin reuptake inhibitors were the first 3 prescribed drugs in our study. Studies recommend the use of benzodiazepines (midazolam, lorazepam, diazepam, clonazepam) in short-term anxiety treatment, and selective serotonin reuptake inhibitors, serotonin and norepinephrine reuptake inhibitors, pregabalin and, if necessary, antipsychotics in long-term treatment. Drug interactions, side effects (dependence, confusion, falls, etc), and the speed of onset of action should be taken into consideration.^[29,50]^ Low-dose antipsychotics are used in cases where rapid effects are desired due to difficulties in access to benzodiazepines; also, selective serotonin reuptake inhibitors are used in long-term treatment since benzodiazepines cause risks such as delirium, falls, confusion, and respiratory depression. In addition, since this study was conducted by examining psychiatric consultations, benzodiazepine use may be more frequent in agitated and anxious patients by palliative specialists.

Risperidone, olanzapine, quetiapine, and aripiprazole are recommended for treatment in patients with psychotic episodes; among these drugs, drug/dose selection should be decided according to the drug side effect profile, patient’s disease, comorbidities, age, and physical condition.^[51]^ Among the reasons why risperidone was most frequently used in our study, it is easy to access due to its availability in our hospital pharmacy, it is a potent antipsychotic, and psychiatrists are accustomed to using it.

This study has several limitations. Primarily, being a single-center, retrospective, and cross-sectional design restricts the generalizability of the findings, as the patient sample may not fully represent the wider population. Although the overall sample size was calculated through an a prior power analysis and found to be statistically sufficient, certain methodological constraints still exist. In particular, the subgroup distribution especially for age was imbalanced, with a relatively small number of patients aged 18 to 64 compared to those aged ≥65. This imbalance may have reduced the statistical power for age-related comparisons and increased the risk of type II error. Moreover, multiple subgroup comparisons were performed, which may have increased the likelihood of type I error. Therefore, these subgroup findings should be interpreted with caution and validated in larger, multi-center prospective studies. Secondly, the study findings were obtained from the consultation notes of psychiatrists, not from the tests performed on the patients. This means that the diagnoses made according to the clinical opinions of psychiatrists may vary, and since standardized scales/structured clinical interviews were not used for diagnosis, the data may not be standardized. Thirdly, since not all patients were screened and the consultations included patients consulted by PCU physicians, the data may not reflect the entire population, and moreover, it may have caused the situations that PCU physicians considered important/could recognize to come to the fore. Lastly but not least, the priority use of drugs available in the hospital for treatment and the difficulties in prescribing/reimbursing/accessing some drugs (e.g., benzodiazepines- especially lorazepam, methylphenidate, ketamine, etc) may have affected the choice of treatment.

## 5. Conclusion

The focus of this study is to include psychiatrists in PCU team or communicating with them to ensure effective treatment for diagnoses such as sleep disorders, delirium, anxiety disorders, and depression, which are commonly encountered in PCU units and can be interpreted as early signs of worsening patient prognosis. In addition, educating the PCU team (physician, nurse, auxiliary staff) and caregivers on the clinical manifestations (e.g. agitation, fatigue, etc) of these diagnoses will contribute to the survival and comfort of patients. In the future, multicenter studies with standardized data obtained from screenings at the beginning of hospitalization and at certain intervals with valid scales will provide more solid suggestions/contributions to this field.

## Acknowledgments

We thank statistician Nazmiye Kursun for her support in the statistical analysis of the study.

## Author contributions

**Conceptualization:** Emre Vuraloglu.

**Data curation:** Emre Vuraloglu.

**Investigation:** Emre Vuraloglu.

**Methodology:** Emre Vuraloglu.

**Project administration:** Emre Vuraloglu.

**Resources:** Emre Vuraloglu.

**Supervision:** Mehmet Atilgan.

**Validation:** Emre Vuraloglu, Mehmet Atilgan.

**Visualization:** Emre Vuraloglu, Mehmet Atilgan.

**Writing – original draft:** Emre Vuraloglu, Mehmet Atilgan.

**Writing – review & editing:** Emre Vuraloglu, Mehmet Atilgan.
